# Rules for the Surgeon

**Published:** 1896-04

**Authors:** Horace T. Hanks


**Rules for the Surgeon** to observe in order to prevent the absorption of poison during operations on septic patients:

1. After the hands and arms are made aseptic, dip them in strong ammonia water, or in a saturated solution of oxalic acid. This procedure will instantly reveal to the surgeon the least abrasion of the skin from any cause.

2. All small abrasions, or separations of continuity of skin, should be painted with flexible collodion, and immediately covered with a few fibres of absorbent cotton. Dry this dressing quickly with heat from alcohol lamp, and again paint with flexible collodion, and dry in the same manner. Then sterilize finger in 1 to 100 bichloride solution.

3. If the wounds are on the joints, apply a strip of adhesive plaster over the cotton and collodion dressing, passing the plaster quite around the finger, at least twice. Fasten this dressing securely with thread. Or, instead of the adhesive plaster, draw on a rubber cot or glove. Sterilize finger or hand and dressihg in 1 to 100 bichloride solution.

4. If the hand or finger is wounded during an operation, stop long enough to place on the wound a drop of saturated solution of carbolic acid, or lysol, or creolin, or touch it with a nitrate of silver point. Cover the wound with a small pledget of absorbent cotton, well saturated with carbolized or creolin water, and cover this cotton thoroughly with adhesive plaster. Fasten this plaster securely with thread. Sterilize the finger and dressing by immersing it in 1 to 100 bichloride solution, and proceed with operation.

5. Remember that your health is, or should be, as valuable as the patient's, and that if you have a good assistant to watch the patient, five minutes' time given to dressing your own wound will make no appreciable difference in the result of the operation you are performing.—*Horace T. Hanks.*

